# Obstructive sleep apnoea is independently associated with the metabolic syndrome but not insulin resistance state

**DOI:** 10.1186/1475-2840-5-22

**Published:** 2006-11-01

**Authors:** A Gruber, F Horwood, J Sithole, NJ Ali, I Idris

**Affiliations:** 1 Sherwood Forest Hospitals NHS Trust, Nottinghamshire, Mansfield, UK; 2 Trent RDSU, University of Nottingham, Nottingham, UK

## Abstract

Obstructive sleep apnoea (OSA) is a cardio-metabolic disorder. Whether metabolic syndrome (MS), insulin resistance (IR) and albuminuria are independently associated with OSA is unclear, but defining the interactions between OSA and various cardiovascular (CV) risk factors independent of obesity facilitates the development of therapeutic strategies to mitigate their increased CV risks. We prospectively recruited 38 subjects with OSA and 41 controls. Anthropometric measurements, glucose, lipids, insulin and blood pressure (BP) were measured after an overnight fast. IR state was defined as homeostasis model assessment (HOMA) value >3.99 and MS diagnosed according to the International Diabetes Federation (IDF) criteria. Subjects with OSA were more obese, more insulin resistant, more hyperglycaemic, had higher Epworth score (measure of day time somnolence) and systolic blood pressure levels. The prevalence of MS was higher in OSA compared with non-OSA subjects (74% vs 24%, p < 0.001). The prevalence of microalbuminuria in both groups was negligible. Logistic regression adjusted for age, BMI and smoking showed that the patient with OSA was 5.9 (95% CI 2.0–17.6) times more likely to have MS than non-OSA patient. Triglyceride (p = 0.031), glucose (0.023) and Epworth score (0.003) values were independently associated with OSA after adjusting for BMI and other covariates whilst IR status was found not to be significant. Using the ROC curve analysis, we found that a waist circumference of >103 cm would predict MS in patients with OSA at 75–78% sensitivity and 61–64% specificity. The agreement between MS and IR state in this cohort is poor. Thus, OSA is associated with MS independent of obesity predominantly due to increased triglyceride, glucose and Epworth score values but not IR or microalbuminuria status. This observation suggests an alternative pathogenic factor mediating the increased cardiovascular risk in patients with OSA and MS, other than that due to IR. The independent link between Epworth score and MS in patients with OSA implicates the role of daytime sleepiness and chronic hypoxia as a potential mediator. Given the discordant between MS and IR state, measurement of waist is useful for predicting mainly MS but not insulin resistance status in patients with OSA. Appropriate pharmacological intervention targeting these independent factors is important in reducing the increased CV risks among patients with OSA.

## Background

Obstructive Sleep Apnoea (OSA) has been linked with increased cardiovascular disease (CVD) [1,2]. While obesity *per se *was previously thought to confer independent vascular risk, more recent studies have provided strong evidence that OSA itself is associated with hypertension [3], dyslipidaemia [4] and impaired glucose tolerance [5], independent of obesity. These proatherogenic factors when clustered together in the same individual are known as the Metabolic Syndrome [6].

Insulin resistance has been implicated as central to the pathogenicity of the Metabolic Syndrome [6]. Because central obesity is strongly associated with insulin resistance and generalised endothelial dysfunction [7], waist circumference has been adopted as a surrogate for an individual's insulin resistance status and risk of CVD. Studies on the relationship between insulin resistance and OSA however have yielded conflicting results, with some studies showing significant associations between the two [8,9] while others have not [10,11]. Similarly, studies looking at the effect of treatment of OSA on insulin resistance have also produced contradictory reports, e.g. continuous positive airway pressure (CPAP) treatment improves insulin resistance in one [12] but not in another study [13]. There is also considerable doubt whether subgroup of patients with metabolic syndrome are indeed insulin resistant [14]. In addition, neck circumference rather than waist circumference has been previously reported to be the most important predictor of OSA amongst all anthropometric variables studied [11]. Thus, the associations between the metabolic syndrome, insulin resistance state and central obesity may not necessarily apply among patients with OSA. It also remains unclear if microalbuminuria, a marker of generalised endothelial dysfunction is more prevalent among patients with OSA. Increased understanding on the independent associations between OSA, metabolic syndrome, insulin resistance and microalbuminuria status is important in order to develop appropriate therapeutic strategies to reduce the high cardiometabolic risks in patients with OSA.

The purpose of this study was therefore to: (i) investigate the independent associations between OSA, metabolic syndrome and insulin resistance, (ii) determine if waist and neck circumference could be used to predict the presence of metabolic syndrome and insulin resistance among patients with OSA and (iii) assess the prevalence of microalbuminuria in patients with OSA.

## Methods

### Subjects and measurements

Consecutive subjects referred to the sleep laboratory at the Sherwood Forest Hospitals NHS Trust for suspected sleep apnoea were recruited. Subjects who were taking glucose lowering agents, lipid lowering treatment or those with evidence of significant renal, liver and cardiac disease were excluded. Because of the many diagnostic criteria available for metabolic syndrome, power calculation was derived from systolic and diastolic blood pressure values (a universal component for all metabolic syndrome criteria) previously reported for OSA patients. A sample size of 32 patients per group are required to detect a 10-mmHg difference in systolic blood pressure and a 5-mmHg difference in diastolic blood pressure with a power of 80% (β error = 0.2), α = 0.05^10^. A total of 79 patients were included in the study (38 patients with newly diagnosed OSA and 41 subjects with no OSA). All subjects underwent an in-patient sleep study assessment. A questionnaire on demographics, medical history, medications, lifestyle and physical activity was completed prior to their study. Daytime sleepiness was assessed using the Epworth sleepiness score. Body habitus was measured in light clothing using standard athropometric methods. Waist circumference was measured midway between the lower costal margin and the iliac crest and neck circumference at the level of the laryngeal prominence. Blood pressure was taken in the sitting position using Dinamap (Critikon Inc, Florida) after 5 minutes of rest. Venous blood was obtained in the fasting state on the morning after the sleep study for the measurement of glucose, insulin, lipids and other routine biochemistry. Mid stream urine (MSU) was analysed for the presence of microalbuminuria using the albumin-creatinine ratio. All subjects gave written informed consent to the study. The study was approved by the local research ethics committee.

### Sleep study assessment

All patients underwent a standard sleep study, using the minimal patient contact sleep diagnosis system (VISI-3, Stowood Scientific Instruments Ltd (SSI), Oxford), which included the following parameters: digital Video/audio, calibrated sound level measurement, electrocardiography, R-R timing, pulse transit time, airflow, respiratory effort, body position and movement. Oxyhaemoglobin saturation (SaO_2_) was measured by a pulse oxymetry. A software package was used for downloading, viewing, reporting and analysis of trend recording oximeters and capnometers. Continuous nasal airflow delivery was by Horizon Nasal CPAP system.

### Insulin resistance and metabolic syndrome assessment

We used homoestasis model assessment (HOMA-IR index) as a measure of insulin sensitivity [plasma glucose (mol/l) × plasma insulin (mU/l)/22.5] – an established test in epidemiological studies. We defined insulin resistance as a HOMA score >3.99, on the basis of a definition for a white population [15]. The Metabolic Syndrome is diagnosed according to the recent IDF guidelines [16], i.e. central obesity, defined as waist equal to or more than 94 cm for males and 80 cm for females; plus two of the following: triglycerides >1.7 mmol/L; HDL-cholesterol <1.04 mmol/L in males and <1.29 mmol/L in females; blood pressure >130/85 mm Hg or use of antihypertensive agent; and fasting hyperglycemia, defined as glucose = or >5.6 mmol/L or previous diagnosis of diabetes or impaired glucose tolerance.

### Statistical analysis

Normally distributed data were presented as means ± standard error mean (SEM), skewed data as the median (ranges) and categorical data as percentage. In this cohort of patients who are significantly obese, gender differences in weight, waist, blood pressure and BMI are negligible. Analysis for both males and females were therefore performed together where appropriate. Univariate comparison of clinical parameters between OSA and non OSA subjects were made using the unpaired t-test or Mann-Whitney test and categorical variables were compared using the Chi-square test. To determine whether OSA is associated with insulin resistance state, metabolic syndrome and individual CVD risk factors independent of obesity, a univariate and multivariate regression analysis (adjusted for age, BMI and smoking history) was performed. Where we had the dependent variable being categorical we used the logistic regression technique. Fasting insulin, HOMA, fasting triglyceride and HDL values were logarithmically transformed before they were used as dependent variables to satisfy the normality requirement. A kappa test (κ-test) was used to assess the agreement between metabolic syndrome and insulin resistance status for individual patients within the cohort. A receiver operating characteristics (ROC) curve was used to select an appropriate cut-off for waist, neck circumferences and Epworth score values that would independently predict likelihood of insulin resistance state and metabolic syndrome status among patients with and without OSA.

## Results

The demographics of our study group are summarised in table 1. In this unadjusted analysis, subjects with OSA had a greater BMI, waist circumference, systolic blood pressure, fasting glucose, fasting triglyceride, fasting insulin, HOMA and Epworth score levels compared with non OSA subjects. The prevalence of the metabolic syndrome was significantly higher in the OSA group compared with non OSA subjects, (73% v 37%, p < 0.001). Other metabolic characteristics included were not significantly different between the two groups. The prevalence of microalbuminuria in both groups was negligible.

**Table 1:** Comparison of clinical and biochemical characteristics in patients with or without obstructive sleep apnoea

	non OSA (n = 41)	OSA (n = 38)	p
Age (years)	47.3 ± 1.7	51.0 ± 2.0	0.17
BMI (kg/m^2^)	30.9 ± 1.1	35.8 ± 1.5	0.01
Waist (cm)	102.1 ± 2.4	114.4 ± 3.0	0.02
Neck (cm)	40.7 ± 0.7	42.7 ± 1.1	0.12
Smoking (%)	17.7	29.7	0.12
Epsworth score	9.51 ± 0.7	13.7 ± 0.9	<0.001
Systolic BP (mmHg)	134.2 ± 2.8	147.1 ± 3.8	0.009
Diastolic BP (mmHg)	82.0 ± 1.7	82.8 ± 2.3	0.784
Total cholesterol (mmol/l)	5.3 ± 0.2	5.2 ± 0.2	0.770
LDL-cholesterol (mmol/l)	3.2 ± 0.2	3.0 ± 0.2	0.308
HDL-cholesterol (mmol/L)	1.3 ± 0.05	1.2± 0.05	0.118
Triglyceride (mmol/l)	1.7 ± 0.2	2.3 ± 0.2	0.010
Glucose (mmol/L)	5.1 ± 0.1	5.9 ± 0.3	0.005
Insulin (pmol/L)	13.7 ± 2.0	21.5 ± 3.3	0.049
HOMA IR	3.1 ± 0.4	6.4 ± 1.3	0.019
Microalbuminuria (%)	6.7	10.8	N/A
Metabolic syndrome (%)	37	73	<0.001

Table 2 summarises independent associations between various demographic and biochemical profiles with the metabolic syndrome and insulin resistance status. After adjustment for age, BMI and smoking history, followed by stepwise multiple regression analysis, only fasting glucose, fasting triglyceride and Epworth score levels were independently associated with a diagnosis of OSA. Based on this analysis, we found that the presence of OSA was independently associated with a nearly six-fold increased risk of having the metabolic syndrome, (OR = 5.88, 95% CI 1.96–17.63, p = 0.002). No independent association was found between OSA and insulin resistance state (OR = 0.54, 95% CI 0.54–1.64, p = 0.3) (Table 3). Because waist circumference is strongly correlated with BMI (r = 0.86), the independent association between OSA and metabolic syndrome persists when adjusted for waist circumference (p = 0.002). Using κ-test, we found that the agreement between subjects' insulin resistance state and the metabolic syndrome status for the study cohort was poor (κ = 0.31, 95% CI 0.09–0.52).

**Table 2:** Multiple regression analysis looking at the independent associations between obstructive sleep apnoea with cardiovascular and anthropometric parameters after adjustment for age, BMI and smoking history. t and p values corresponds to OSA v non OSA status.

VARIABLES	t-value	p-value
Systolic blood pressure	-1.83	0.071
Diastolic blood pressure	0.34	0.737
Waist	-0.85	0.398
Neck	-0.46	0.646
Total cholesterol	0.09	0.931
LDL cholesterol	0.66	0.511
HDL cholesterol*	0.97	0.337
Triglyceride*	-2.19	0.031
Glucose	-2.32	0.023
Insulin*	-0.71	0.482
Epworth score	-3.05	0.003

* log transformed prior to analysis

**Table 3:** Independent associations between obstructive sleep apnoea and metabolic syndrome or insulin resistance status (after adjustments for age, BMI, smoking history)

**Parameters**	**P**	**Odds ratio (confidence interval)**
Metabolic syndrome	0.002	5.88 (95% CI 1.961–17.63)
Insulin resistance	0.279	0.54 (95% CI 0.544–1.642)

**Figure 1:**
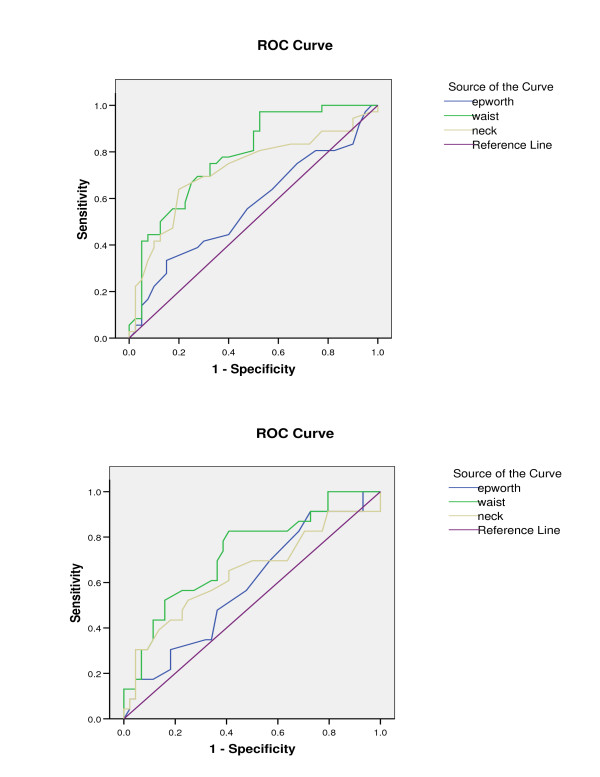
ROC curve analysis to determine cut off values for waist and neck circumference that would predict (a) metabolic syndrome and (b) insulin resistance.

On the basis of the ROC curves analysis, we found that an optimal waist circumference cut-off for detecting metabolic syndrome to be at 103 cm. Sensitivities and specificities for this cut off level were between 75–78% and 61–64% respectively. For neck circumference, an optimal cut off of 42 cm was set for men, with sensitivities and specificities of 78% and 60% respectively. No suitable neck circumference cut off was found for women (Figure 1). Using similar analysis, we set the optimal cut-off for detecting insulin resistance state at 105 cm for waist circumference in both sexes (sensitivity and specificity of 78% and 62% respectively). No suitable neck circumference cut off value could be used for detecting the presence of insulin resistance state. Similarly, Epworth score criteria could not be used to detect the presence of metabolic syndrome or insulin resistance state in this cohort.

## Discussion

In this hospital based cohort, OSA was shown to be independently associated with the metabolic syndrome (IDF criteria), driven largely by increased serum triglyceride, elevated glucose and day-time sleepiness but not with insulin resistance state. The agreement between subjects' metabolic syndrome and insulin resistance status is poor. These findings suggest that in patients with OSA, other mechanisms other than insulin resistance may play a bigger role in the pathophysiology of the metabolic syndrome. Waist circumference could be used to help identify patients with metabolic syndrome, whereas neck circumferences showed no appropriate cut off values. Our observations enhance findings of previous studies and expand knowledge of the clinical utility of measuring waist circumference for detecting metabolic syndrome and insulin resistance. The study have also provided important information regarding appropriate targets for therapeutic intervention among patients with OSA in order to reduce their cardio-metabolic risks.

Insulin resistance is generally believed to play a central role in the clustering phenomenon of CVD risk factors that defines the metabolic syndrome [6]. The relationship between insulin resistance and OSA however is somewhat unclear [8-13]. Discrepancies in study outcomes may be explained by differences in study population or methodologies but a more likely explanation is that the mechanistic links between the various metabolic and biological mechanism of increased CVD risks in patients with OSA is one of complex interaction involving paradoxical rises in blood pressure, cortisol levels and sympathetic activity during sleep mediated by stress, hypoxia and increased free fatty acid lipolysis [17,18]. The interpretation of *in-vivo *metabolic studies is further complicated because hypoxia inhibits insulin secretion through decreased β-cell ATP production [19] but may also reduce insulin receptor tyrosine kinase activity leading to insulin resistance and hyperinsulinaemia [20]. Although CPAP treatment has been shown to improve HbA1c [21] and HDL-cholesterol [22] levels in patients with OSA, the increasingly reported associations between diabetes and sleep disordered breathing, in terms of cause or effect [23,24], has provided clinicians with a wider metabolic targets potentially amenable to pharmacological and lifestyle interventions. Observation from this study however does not support the potential use of insulin sensitizer among patients with OSA.

There is also evidence to suggest that OSA is independently associated with increased activation of the renin-angiotensin-system [25] – an important correlate of cardiovascular and metabolic risks. In support of this, a recent study showed that abdominal adiposity is related to the development of elevated albuminuria in both sexes [26]. Owing to the importance of microalbuminuria as a major risk factor for CVD in the general population [26,27], the World Health Organization (WHO) includes microalbuminuria in their criteria for diagnosing the metabolic syndrome [28]. Our study however showed that the prevalence of microalbuminuria, a recognized marker of systemic generalized endothelial dysfunction is negligible in patients with OSA. After adjusting for known covariates including BMI in our study, only raised triglyceride and fasting glucose were independently associated with OSA. These findings are important when considering pharmacological intervention for primary CVD prevention in patients with OSA.

Because abdominal obesity is recognized to be a better predictor of CVD than BMI [7,29], waist circumference has been adopted to reflect individual's CVD risks. We found that a waist circumference of >103 cm to be an important predictor for metabolic syndrome state. This cut point value concurs with findings from a study by Laaka et al [30], who showed that CVD and overall mortality was more consistently increased using a waist circumference criterion of 102 cm rather than the 94 cm adopted by the IDF and the WHO to diagnose the metabolic syndrome. While previous studies have shown that neck circumference was the most important predictor of OSA among all athropometric variables, observation from our study did not support the use of neck circumference for predicting metabolic syndrome or insulin resistance status and concurs with finding from a more recent study using nuclear magnetic resonance imaging (NMR) [31].

Several limitations of our study must be acknowledged. This study was not designed to include matched control subjects. Because the aims were to determine independent associations between OSA, metabolic syndrome, insulin resistance status and abdominal obesity, we included all patients who fulfilled inclusion criteria to avoid selection bias. There might also be confounding of metabolic and haemodynamic parameters due to use of antihypertensive drugs. This confounder is likely to be not significant because the number of patients taking antihypertensive drugs is small. Finally, although euglycaemic hyperinsulinaemic clamp is generally regarded as the gold standard for measuring insulin sensitivity, HOMA-IR is a simpler, inexpensive technique and has shown strong correlation (r = 0.82) when validated against the insulin-clamp technique [32].

In conclusion, OSA is independently associated with the metabolic syndrome but not insulin resistance state. The observed independent association is largely driven by fasting glucose and triglyceride levels. The agreement between metabolic syndrome and insulin resistance status is poor in this cohort, which concurs with studies from a more general population cohort [33]. The biological mechanism of increased CVD risk in patients with OSA is therefore likely to be multifactorial which includes the parameters mentioned above and possibly hypoxia mediated. Pharmacological interventions for patients with OSA should therefore include strategies to lower serum triglyceride, glucose in addition to lifestyle intervention and CPAP therapy. Waist circumference measurement could be a used to predicting metabolic syndrome and possibly CVD risk in patients with OSA.

## Competing interests

The author(s) declare that they have no competing interests.

## Authors' contributions

AG and FH was involved in study design, coordination and data acquisition, JS performed the statistical analysis, NJA participated in the design of the study and data interpretation. II conceived the study, and participated in its design, coordination and drafted the manuscript. All authors read, contributed towards and approved the final manuscript.
